# Supplementary material for: The effects of genital myiasis on the diversity of the vaginal microbiota in female Bactrian camels
Source: BMC Vet Res. 2022 Mar 5;18:87. doi: 10.1186/s12917-022-03189-5 (PMC8897907; doi:10.1186/s12917-022-03189-5)
Supplement: Supplementary file 5 — Additional file 5. [file 12917_2022_3189_MOESM5_ESM.zip › MPL201709200_16s_yy/Treat1/B13_bdiv/unweighted_unifrac.pdf]

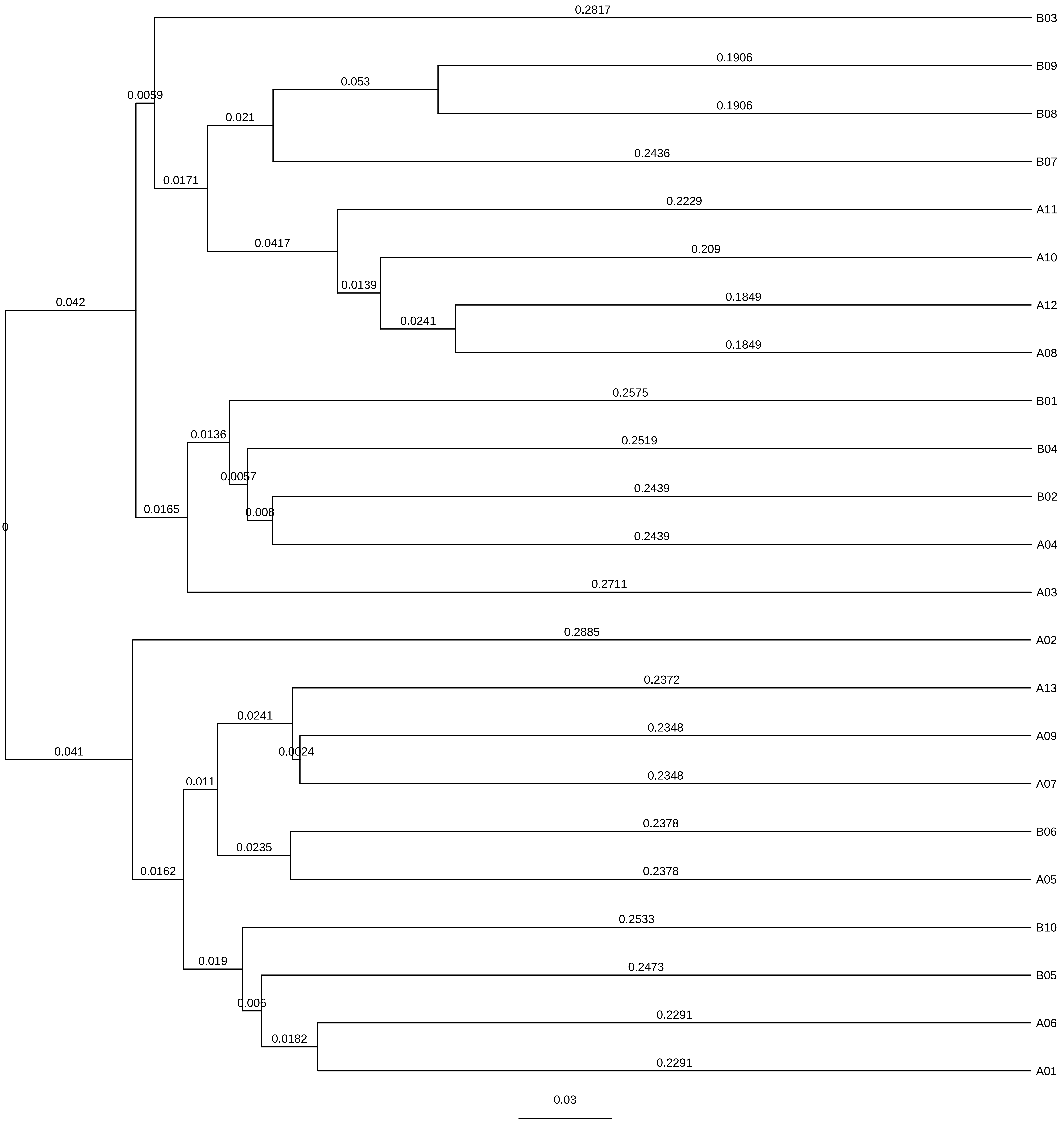
0.2817
B03
0.1906
B09
0.053
0.0059
0.1906
B08
0.021
0.2436
B07
0.0171
0.2229
A11
0.0417
0.209
A10
0.0139
0.1849
A12
0.042
0.0241
0.1849
A08
0.2575
B01
0.0136
0.2519
B04
0.0057
0.2439
B02
0.0165
0.008
0
0.2439
A04
0.2711
A03
0.2885
A02
0.2372
A13
0.0241
0.2348
A09
0.0024
0.041
0.2348
A07
0.011
0.2378
B06
0.0235
0.0162
0.2378
A05
0.2533
B10
0.019
0.2473
B05
0.006
0.2291
A06
0.0182
0.2291
A01
0.03
